# The Professional Identity and Career Attitude of Chinese Medical Students During the COVID-19 Pandemic: A Cross-Sectional Survey in China

**DOI:** 10.3389/fpsyt.2022.774467

**Published:** 2022-02-15

**Authors:** Xingjie Yang, Lan Gao, Suoyuan Zhang, Libin Zhang, Ligang Zhang, Shuangjiang Zhou, Meng Qi, Jingxu Chen

**Affiliations:** ^1^Beijing HuiLongGuan Hospital, Peking University HuiLongGuan Clinical Medical School, Beijing, China; ^2^Collaborative Innovation Center of Assessment for Basic Education Quality, Beijing Normal University, Beijing, China; ^3^Department of Applied Psychology, Chengde Medical College, Chengde, China

**Keywords:** professional identity, career attitude, COVID-19 pandemic, China, medical students, health emergency

## Abstract

**Background:**

Although professional identity is a strong predictor of career choice, only a few studies have reported on medical students' career attitude during a public health emergency. This study investigates the changes in medical students' professional identity and career attitude during the COVID-19 pandemic, evaluates their mental health and social support system under stress, and explores the relationship between their career attitude and other factors.

**Methods:**

An online survey of 6,226 Chinese medical students was conducted to collect information on demographics, professional identity, and career attitude. The collected data were assessed using the Patient Health Questionnaire, the Generalized Anxiety Disorder Scale, and the Social Support Rating Scale.

**Results:**

The results revealed that most (80.8%) of the participants did not change their career attitude and the professional identity of most participants strengthened, and they preferred to participate on the frontline during the COVID-19 pandemic. The prevalence of depression and anxiety among medical students was 22.86% and 35.43%. Low social support, depressive symptoms, male gender, and higher grades were factors that negatively affected career attitude.

**Conclusions:**

After the outbreak of the pandemic, it was necessary to conduct diversified professional identity research to support medical students, especially those with low social support and depressive symptoms.

## Background

COVID-19 is considered a global pandemic and has been raging since March 11, 2020 (1), seriously threatening the health of people worldwide. In many countries, there was a serious shortage of medical workers because of the increased demand for professionals to help; consequently, retired doctors were recalled, and medical students were sent to help in the fight against the pandemic. In China, more than 42,600 medical workers went to the most seriously affected areas to help (2). In this sudden public health crisis, doctors renewed their sense of value and honor in the profession. Medical students are a major component of medical reserve forces and must also participate in ensuring the health and safety of the population (3). Therefore, it is important to assess their professional identity and psychological state during the pandemic.

Under stressful situations, individuals have different emotional, cognitive, and behavioral responses. An individual's cognitive evaluation also affects their stress responses (4). The COVID-19 pandemic has had profound mental health consequences for many people (5), especially college students (6). A previous study indicated that medical University students experienced poor mental states during the COVID-19 outbreak (7).

One's professional identity is an individual's professional self-concept based on their beliefs, values, motives, attributes, and experiences (8) and is derived from and perceived in terms of the role that individuals assume in their work. Professional identity is a factor affecting job satisfaction (9). It is an important part of nurturing professionalism among medical students (10, 11) and is related to how strongly individuals identify with their careers. Some scholars also pay attention to the formation and factors that influence medical students' professional identity (12–15). For physicians-in-training, preliminary data suggest that good virtues in medical practice are associated with a strong sense of professional identity (16). Further, career attitude refers to the tendency of medical students to pursue the medical profession in the future; It depends upon different interlinked factors (17) and formed by a matching of perceptions of specialty characteristics with personal needs (18), such as expected salary, intellectual satisfaction, workload, experience during the medical schools, the student values and professional attitude, and so on. Professional identity is an important predictor of continuing to do the job (9). Previous studies have also shown that students' career attitude would be affected by their mental health state (19), depressive symptoms were considered predictors for professional exhaustion which would affected career attitude (20).

Recently, the professional identities of medical students have gradually studied. During the COVID-19 pandemic, some scholars paid attention to the career attitudes (21, 22) and willing to volunteer (23) in medical students; medical students are motivated by a sense of purpose or duty, altruism, perception of good performance and values of professionalism. Previous studies also have focused on the tense physician-patient relationship and violent injuries in China, which may have affected medical students' professional identity (24). To the best of our knowledge, the professional identity and career attitude of medical students in China during the COVID-19 pandemic have yet to be investigated. Therefore, this study has attempted to investigate the changes in medical students' professional identity and career attitude during the COVID-19 pandemic, evaluate their mental health and social support system under stress, and explore the relationship between their career attitude and correlated factors.

## Methods

### Participants

We used an online survey to conduct a cross-sectional study on the professional identity and mental health of medical students from February 11 to 19, 2020. All data were collected online via a self-reported questionnaire using the Wenjuanxing platform (https://www.wjx.cn/). Participants were recruited with a snowball sampling method through wechat and social media in the form of Wenjuanxing. Participants were encouraged to forward the link to other relevant respondents. Prior to filling the questionnaire, participants were informed that they had the rights to withdraw their consents at any time, and that all information would be kept anonymous and confidential throughout the study. Inclusion criteria were full-time medical University students, including undergraduate from grade 1 to grade 5, living in mainland China, ≥18 years of age. A total of 6,318 participants took part in the survey. After excluding incomplete questionnaires and those that were completed in <3 min, 6,226 participants from 31 provinces and autonomous regions were included in the analysis.

Approval for the study was obtained from the Ethics Committee of Beijing HuiLongGuan Hospital. All participants provided informed consent online to participate in the study.

The demographic section was designed by the research team to collect the general characteristics of medical students, including gender, age, grade, hometown, and 2019-nCoV exposure (2019-nCoV exposure means being diagnosed with COVID-19 or having a history of close contact).

### Assessment of Professional Identity

The questionnaire was designed to evaluate six factors (professional cognition, professional emotion, professional commitment, professional behavior, professional achievement, and professional value) of professional identity after consulting the relevant literature (25). One item was selected from each of the six dimensions of medical students' professional identity scale (25), and a simple medical students professional identity scale (see Appendix A) was developed to evaluate professional identity. The response for each item consists of five choices: from 1 (strongly disagree) to 5 (strongly agree). In this study, Cronbach's α was 0.857 and 0.890 before and after the pandemic, respectively.

### Assessment of Career Attitude

Evaluation of career attitude was conducted by assessing medical students' attitude after the pandemic (the following question was asked: “Did your willingness to practice medicine change after the pandemic?” 1. unchanged; 2. enhanced; 3. weakened). Based on the results, we divided medical students into three groups: unchanged, enhanced, and weakened.

### Assessment of Reasons of Studying Medicine

We reviewed the literatures (17, 18) and listed the reasons why medical students were willing or unwilling to continue studying medicine, the reasons were allowed multiple option. Students choose from the seven options below: The reasons for the willingness to be a doctor include: (1) “Being a doctor is my dream.” (2) “Doctors are respected.” (3) “Doctors are valuable.” (4) “Doctors are paid well.” (5) “Doctors have rich social connections.” (6) “After the outbreak, the state will provide more support to doctors.” (7) “Nothing else matters.” The reasons for not being willing to be a doctor include: (1) “Being a doctor is stressful.” (2) “The workload of doctors is too heavy.” (3) “An outbreak of infectious disease increases the risk to doctors and their families.” (4) “Doctor-patient relations are strained.” (5) “Doctors are poorly paid.” (6) “I did not like studying medicine.” (7) “I have other career options.”

### Assessment of Depressive Symptoms

Depressive symptoms were screened using the 9-item Patient Health Questionnaire (PHQ-9) (26). The PHQ-9 has been widely used in China, and the reliability and validity of the Chinese version of the PHQ-9 has been demonstrated (27). The PHQ-9 was scored from 0 to 27; Cronbach's α, in this case, was 0.87. A PHQ-9 score > 5 was considered indicative of depressive symptoms.

### Assessment of Anxiety Symptoms

Anxiety symptoms were screened using the 7-item Generalized Anxiety Disorder Scale (GAD-7) (28). The GAD-7 has been widely used in China, and the reliability and validity of the Chinese version of the GAD-7 has been confirmed (29), with scores ranging from 0 to 21. Cronbach's α for this case was 0.92. A GAD-7 score > 5 was considered indicative of anxiety symptoms.

### Assessment of Social Support

Social support was assessed using the Social Support Rating Scale (SSRS) (30), which has already been used widely in various studies in different Chinese communities and has been shown to have good validity and reliability (31) a higher score indicating more social support. Final scores were divided into three grades (high, moderate, and low).

### Analysis

Data analysis was performed using SPSS statistical software (version 24.0; IBM Corp). The chi-square test was used to compare the changes in the career attitude of medical students in different demographic categories. Scores obtained from the GAD-7, PHQ-9, and SSRS for the three groups of medical students were also compared. The rank sum test was used to analyze differences in the dimensions of professional identity among the three groups of medical students before and after the pandemic outbreak. Multivariate disordered logic regression was used to analyze the factors influencing changes in career attitude. The level of significance was set at 0.05 (two-sided).

## Results

A total of 6,226 full-time medical undergraduates aged 18–27 years completed the questionnaires. The median age of the participants was 21 years, of which 60.1% were female. The students came from all provinces of China, except Macau, and 98.6% took the survey at home. A total of 79.0% of the respondents revealed that they would like to participate in the frontline. And 92.0% of them has no 2019-nCoV exposure.

As shown in Table 1, the number of participants who did not change their career attitude was 4,989 (80.1%), while enhanced career attitudes accounted for 741 (11.9%) of the respondents. Students whose career attitude was weakened totaled 496 (8.0%). There were statistical differences among students from different academic years (*P* < 0.05) and hometowns (*P* < 0.05).

**Table 1:** The changes in career attitude among medical students with different socio-demographic characteristics (*N* = 6,226).

**Variables**	***n* (%)**	**Unchanged**	**Enhanced**	**Weakened**	***X^**2**^***	***P***
		**(*n =* 4,989)**	**(*n =* 741)**	**(*n =* 496)**		
Gender					2.78	0.249
Male	2,484 (39.9)	1,969 (79.3)	289 (11.6)	226 (9.1)		
Female	3,742 (60.1)	3,020 (80.7)	452 (12.1)	270 (7.2)		
Hometown					7.299	0.026
Urban resident	2,213 (35.5)	1,763 (79.7)	248 (11.1)	202 (9.1)		
Rural resident	4,013 (64.5)	3,226 (80.4)	493 (12.3)	494 (12.3)		
Grade					19.335	0.013
1st year	1,875 (30.1)	1,494 (79.7)	243 (13.0)	138 (7.3)		
2nd year	819 (13.2)	638 (77.9)	110 (13.4)	71 (8.7)		
3rd year	784 (12.6)	605 (77.2)	104 (13.3)	75 (9.6)		
4th year	1,331 (21.4)	1,083 (81.4)	150 (11.3)	98 (7.4)		
5th year	1,417 (22.7)	1,169 (82.5)	134 (9.5)	114 (8.0)		
2019-nCoV exposure					0.564	0.754
No	5,727 (92.0)	4,594 (80.2)	681 (11.9)	452 (7.9)		
Yes	499 (0.8)	395 (79.2)	60 (12.0)	44 (8.8)		

**Figure 1:**
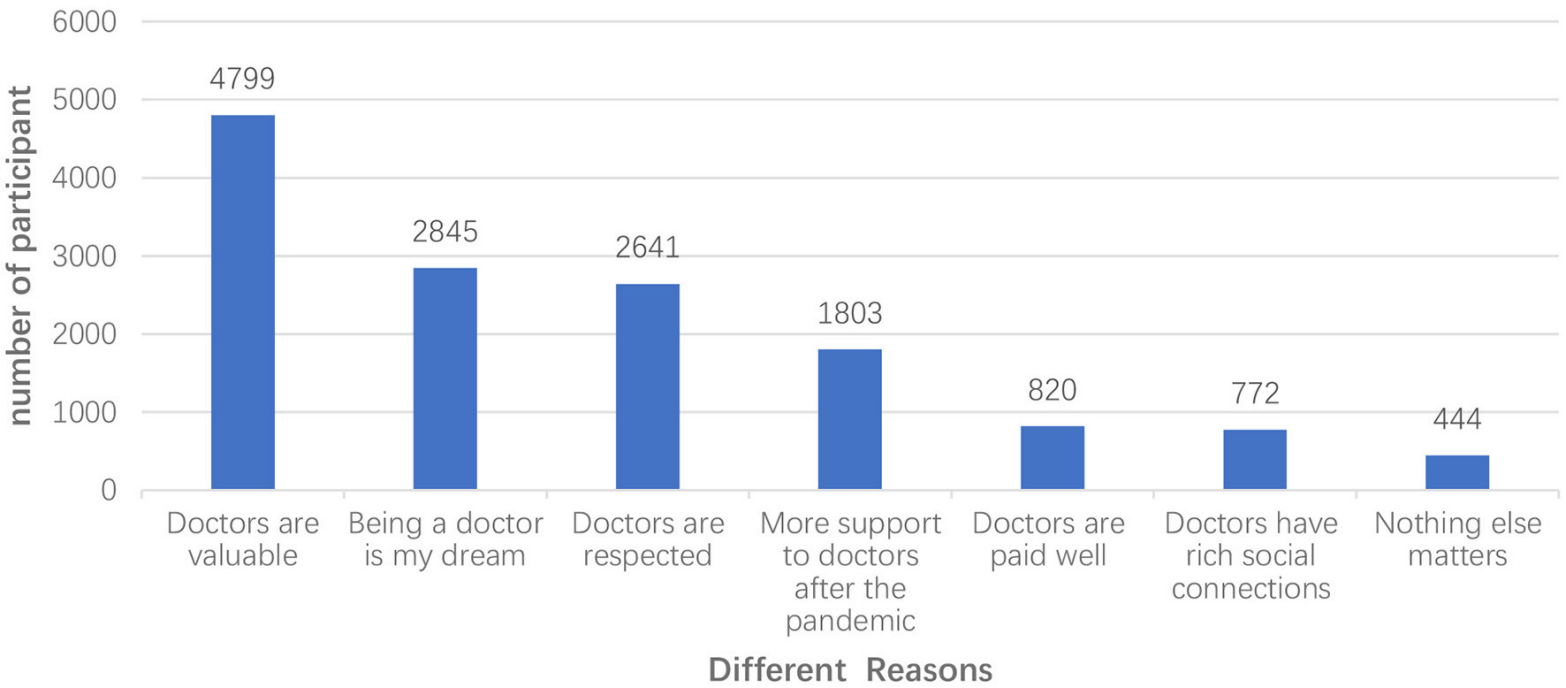
Reasons of willingness to be doctor.

**Figure 2:**
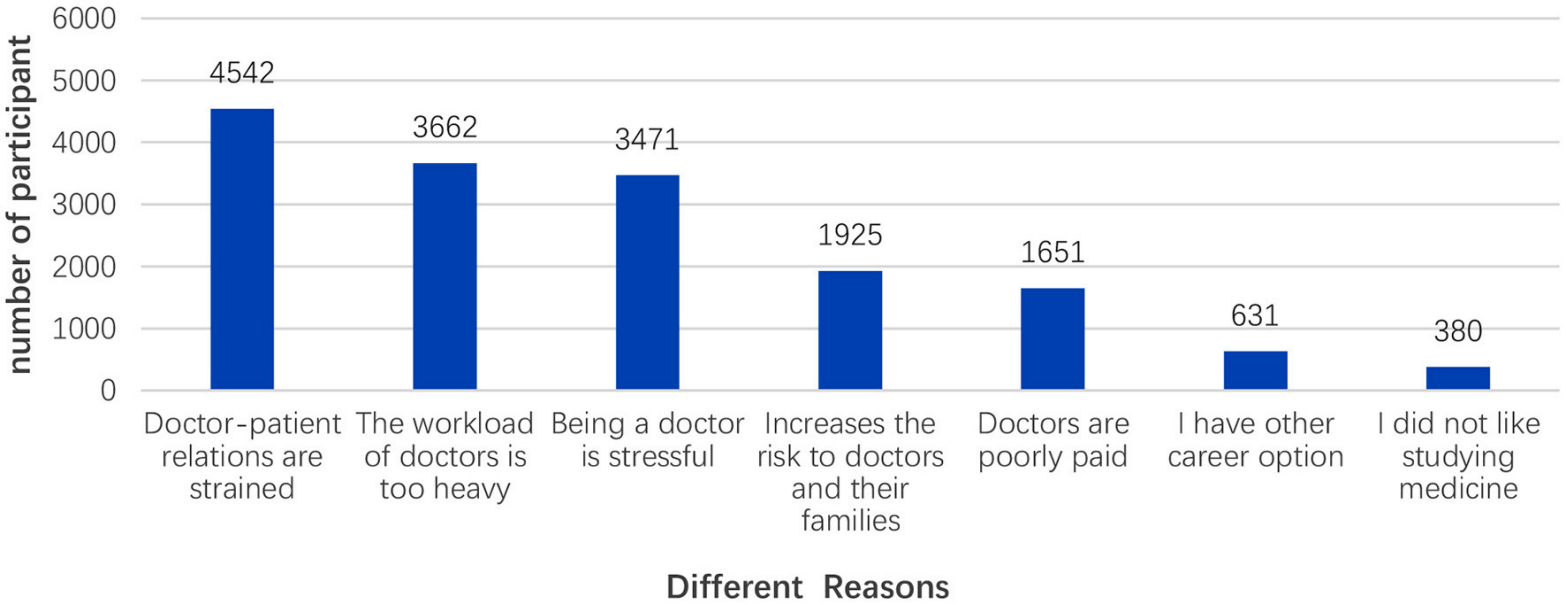
Reasons of unwillingness to be doctor.

We then investigated why medical students were willing and unwilling to become doctors. As shown in Figure 1, the top three reasons for willingness to become a doctor were: (1) “Doctors are valuable.” (2) “Being a doctor is my dream.” (3) “Doctors are respected.” The top three reasons for not being willing to become a doctor were: (1) “Doctor-patient relations are strained.” (2) “The workload of doctors is too heavy.” (3) “Being a doctor is stressful.” (Figure 2).

Table 2 includes the results that were obtained using the non-parametric Wilcoxon signed-rank test. In the enhanced group, there was no significant difference in professional achievement before and after the pandemic (*P* = 0.494); however, the difference was statistically significant for the rest of the groups. In the weakened group, there were significant differences in professional cognition, commitment, achievement, and value.

**Table 2:** The professional identity among medical students with different career attitudes before and after the pandemic (*N* = 6,226).

**Variables**		**Career attitude**		
		**Unchanged**	**Enhanced**	**Weakened**
Professional cognition	Z	−13.911	−8.026	−2.5
	P	0.000	0.000	0.012
Professional emotion	Z	−6.276	−6.633	−1.333
	P	0.000	0.000	0.182
Professional commitment	Z	−3.260	−7.994	−4.926
	P	0.000	0.000	0.000
Professional behavior	Z	−6.791	−7.852	−1.600
	P	0.000	0.000	0.110
Professional achievement	Z	−13.126	−0.685	−6.367
	P	0.000	0.494	0.000
Professional value	Z	−3.121	−6.598	−5.925
	P	0.002	0.000	0.000

Scores (cutoff score 5) from GAD-7 and PHQ-9 tests were used to divide respondents into the “anxiety group” and the “depression group,” Based on the GAD-7 and PHQ-9, the prevalence of depression and anxiety among medical students was 22.86 and 35.43%, respectively. A chi-square test showed that there were significant differences in anxiety, depression, and social support among the three groups of medical students with different career attitude (summarized in Table 3).

**Table 3:** Depressive symptoms, anxiety symptoms and social support among medical students with different changes in career attitude (N = 6,226).

**Variables**	***N* (%)**	**Changes of career attitude**				
		**Unchanged**	**Enhanced**	**Weakened**	***X^**2**^***	***P***
Depressive symptoms				14.747	0.001	
Yes	2,206 (35.4)	1,724 (79.0)	268 (12.1)	214 (9.7)		
No	4,020 (64.6)	3,264 (81.2)	473 (11.8)	282 (7.0)		
Anxiety symptoms				11.088	0.004	
Yes	1,423 (22.9)	1,101 (77.4)	182 (12.8)	140 (9.8)		
No	4,803 (77.1)	3,888 (80.9)	559 (11.6)	356 (7.4)		
SSRS					31.063	0.000
Low	2,151 (34.6)	1,703 (79.2)	227 (10.6)	221 (10.3)		
Medium	2,146 (34.5)	1,731 (80.7)	257 (12.0)	158 (7.4)		
High	1,929 (31.0)	1,555 (80.6)	257 (13.3)	117 (6.1)		

We set the dependent variable of weakened, unchanged, and enhanced groups to 0, 1, and 2, respectively, and then performed an ordered multivariate logistic regression analysis. The parallel line hypothesis test showed *P* < 0.05, which indicated that the data could not be analyzed using this method. Therefore, we conducted a disordered multivariate logistic regression analysis. The resultant chi-square value of the model was 139.49 (*P* < 0.001), which indicated its statistical significance. The pseudo-R2 of Nagelkerke was 0.031, which indicated that the model corresponded, to a certain degree, with the dependent variables (Table 4).

**Table 4:** Multivariate disordered logic regression of the factors associated with the changes in career attitude (*N* = 6,226).

**Career attitude**		**B**	**SE**	**Sig**.	**Exp (B)**	**95% Confidence Interval for Exp (B)**	
						**Lower Bound**	**Upper Bound**
Enhanced	Intercept	−2.579	0.303	0			
	Gender						
	Male	−0.003	0.082	0.971	0.997	0.849	1.17
	Grade						
	1st year	0.318	0.115	0.006	1.374	1.096	1.722
	2nd year	0.372	0.138	0.007	1.451	1.106	1.903
	3rd year	0.371	0.141	0.008	1.450	1.100	1.910
	4th year	0.189	0.127	0.136	1.208	0.943	1.548
	Depressive						
	No	−0.132	0.085	0.120	0.876	0.742	1.035
	SSRS						
	Low	−0.165	0.103	0.109	0.848	0.694	1.037
	Medium	−0.071	0.097	0.460	0.931	0.771	1.125
	Does your family support you to fight the epidemic						
	Strong support	1.014	0.298	0.001	2.755	1.537	4.941
	General support	0.656	0.289	0.023	1.927	1.093	3.399
	Neutrality	0.615	0.284	0.031	1.850	1.059	3.231
	Opposed	0.345	0.307	0.261	1.412	0.774	2.578
	Hometown						
	Urban resident	−0.105	0.084	0.210	0.900	0.763	1.061

*Women, 5th year, people with depressive symptoms, high level of SSRS, family strongly opposed you to fight against the epidemic and rural resident were selected as the reference group*.

Compared to subjects in the unchanged group, students in junior academic classes of medical school were more likely to enhance their career attitudes, whereas the attitude of freshmen was 1.374 times higher than that of fifth-year students. Subjects whose family members strongly supported them in their fight against the pandemic were more likely to enhance their career attitude than those who were strongly opposed by their family members. The number of subjects who were strongly supported by their families was 2.755 times higher than those whose family members were strongly opposed (Table 4).

Compared to subjects in the unchanged group, males were more likely to weaken their career attitude than females, and medical students with depression were more likely to weaken their attitudes than those with high social support. Students who lived in cities were 1.248 times less likely to practice medicine than those living in rural areas. The results are summarized in Table 5.

**Table 5:** Multivariate disordered logic regression of the factors associated with the changes in career attitude (*N* = 6,226).

**Career attitude**		**B**	**SE**	**Sig**.	**Exp (B)**	**95% Confidence Interval for Exp (B)**	
						**Lower Bound**	**Upper Bound**
Weakened	Intercept	−2.070	0.242	0			
	Gender						
	Male	0.225	0.096	0.020	1.252	1.037	1.513
	Grade						
	1st year	−0.002	0.134	0.986	0.998	0.767	1.298
	2nd year	0.167	0.161	0.299	1.182	0.862	1.621
	3rd year	0.269	0.159	0.090	1.309	0.959	1.786
	4th year	−0.101	0.145	0.485	0.904	0.680	1.201
	Depressive						
	No	−0.249	0.099	0.012	0.780	0.642	0.947
	SSRS						
	Low	0.326	0.125	0.009	1.386	1.084	1.771
	Medium	0.103	0.129	0.424	1.108	0.861	1.426
	Does your family support you to fight the epidemic						
	Strong support	−1.116	0.277	0.000	0.328	0.190	0.564
	General support	−0.713	0.221	0.001	0.490	0.318	0.755
	Neutrality	−0.440	0.204	0.031	0.644	0.432	0.960
	Opposed	−0.008	0.220	0.970	0.992	0.644	1.527
	Hometown						
	Urban resident	0.221	0.097	0.023	1.248	1.032	1.510

*Women, 5th year, people with depressive symptoms, high level of SSRS, family strongly opposed you to fight against the epidemic and rural resident were selected as the reference group*.

## Discussion

The career attitude formed by a matching of perceptions of specialty characteristics with personal needs (18) and the characteristics of students such as age, gender, geography and study year (17). In this study, there were statistical differences among students from different academic years and hometowns. Previous studies have shown that the professional identity of medical students decreases over time (32, 33). At the time of our survey, medical students were staying in their home towns on winter vacation. And medical students from urban resident or rural resident are different, this could be explained by imbalances between economy, culture, and different publicity efforts relating to pandemic prevention knowledge between urban and rural areas (21).

In this study, most (80.8%) of the participants did not change their career attitude, and 741 (11.9%) of the total respondents enhanced their career attitude. This shows that the majority of medical students have a strong desire to become doctors, indicating that they have a strong professional identity. Professional identity is an important factor in the development of medical education and practice, and the transformation of medical students' professional identity is at the core of medical education (11). Those who possess a strong professional identity are more likely to be connected to their line of work and find a greater sense of purpose in life through it (34). In addition, medical students' career attitudes are influenced by their cognition about, attitude toward, and evaluation of their future careers. The formation and evolution of a professional identity is a dynamic process. Before and after the pandemic, most dimensions of professional identity of medical students changed. These findings revealed that COVID-19 pandemic could affect the professional identities of medical students. This finding was similar to that of a previous study, which concluded that nursing students' professional identity was enhanced after the outbreak of SARS in Hong Kong, China (35). After the outbreak of COVID-19 pandemic, one study (36) found that about one-fifth of surveyed medical students currently believe that the COVID-19 pandemic will affect their choice of specialty. Another study found that the percentage of middle-school students who wanted to study medicine increased to 55.1%, and 29.8% of middle-school students had changed from unwilling to study medicine to willing to study medicine, which indicated that middle-school students had changed their attitude toward medical work after the outbreak of COVID-19 (37). Another study (23) showed that 48.7% of Indonesian medical students were willing to volunteer, shortage of medical personnel and sense of duty were the main reasons increasing the students' willingness to volunteer. The professionalism of medical staff during the epidemic may also have been an inspiration. Some scholars (38, 39) found that the role of models and mentors had a significant impact on the professional identity of medical students. Passi and Johnson (40) also indicated that positive role modeling by doctors effectively enhanced the transformation of a student into a doctor. In this study, 79.0% of the students were likely to engage in the “battle” against the virus. This could be regarded as their “post-traumatic growth,” which suggests that exposure to critical events could lead to opportunities for growth (41). According to the survey in this study, “being a doctor is valuable,” “being a doctor is my dream,” and “being a doctor is respected” are the most popular reasons medical students choose to become a doctor. The sense of value and achievement of doctors is still the main reason medical students choose to become doctors.

This study also found that medical students with depression were more likely to weaken their attitude toward medicine, which might be due to a sense of uncertainty about themselves. Previous studies (42, 43) have shown that depression is one of the most common health problems among University students, especially among medical students who endured heavy financial burdens and study-induced stress (44). After the outbreak of COVID-19, 37.0% of Chinese University students experienced depressive symptoms and 24.9% experienced anxiety symptoms (37). The mental health of these medical students could be a predisposing factor for burnout during residency or postgraduate training (45). This might also affect their choice of future career.

Similarly, it was found that strong social support enhanced medical students' career attitudes. This study also established that the attitude of medical students' family members toward fighting the pandemic also affected students' attitudes toward medical practice. Generally speaking, people with high social support had better resources; they received more support and help coping with their working environment and were more likely to solve problems and difficulties. As a resource available to individuals, social support played an intermediary role in coping with stress, and those with good social support could cope better with it (46). The availability of social support reduced the odds of mental distress for those who experienced it (47). Degree of social support was found to be negatively correlated with anxiety and depression among residents (44). In the current COVID-19 outbreak, high social support can effectively reduce anxiety and improve self-efficacy and sleep quality in COVID-19 patients' caregivers (48). Students who lived alone or had poor relationships with their partners, classmates, or friends scored higher on the depression and anxiety scales. Thus, for medical students under stress due to the public health emergency, good social support was conducive to a positive psychological state and encouraged them to continue to engage in the medical profession.

In addition, freshmen, sophomores, juniors, and senior medical students were more likely to strengthen their career attitudes than fifth-year medical students. Previous studies have shown that the professional identity of medical students decreases over time (32, 33). Iqbal et al. (49) also indicated that higher-grade University students were more depressed and had poorer mental health. The reason might be that the higher-grade students have higher levels of stress (50), and they were affected by a heavy academic load and encountered setbacks in clinical internship. On the other hand, the lower-grade medical students had just entered medical colleges and had not yet completely started the study of clinical medicine; thus, they expected much from themselves.

Males were more likely to weaken their attitudes toward medicine. Some previous studies (32, 33, 51) concluded that females reported stronger identification than males. This might be related to the fact that males bore more social responsibilities and economic pressure (52, 53) which bring more pressure or their less seeking help and coping strategies.

The most significant advancement of this study was that it conducted the largest survey of professional identity and career attitude of medical students during the COVID-19 pandemic. Simultaneously, this study discussed the demographic factors of people with different career attitudes in depth and assessed factors related to mental health status. Further research in the field should focus on ways to improve medical students' mental health and enhance their professional identity.

However, this study has several limitations. First, the cases were recruited using snowball sampling. We could not weigh this sample to increase representativeness because statistics on national medical students were not available. Second, medical students reported their professional identity before the outbreak, and the retrospective nature of the study might have caused recall bias. Finally, although the data collection process was anonymous, online surveys could not verify the identity of respondents, and self-reporting might have been accompanied by personal biases.

## Conclusions

The COVID-19 pandemic not only effected a crisis but reconstructed the professional identity of medical students. After such a crisis, some medical students' professional identity was enhanced, and they were proud of the profession they had chosen to pursue. However, as illustrated above, this was not the case for all medical students. We should pay more attention to medical students with depressive symptoms, low social support, and higher grades. The utilization of social support by medical students could be strengthened through group coaching, which is an effective method of support (54).

## Data Availability Statement

The raw data supporting the conclusions of this article will be made available by the authors, without undue reservation.

## Ethics Statement

The studies involving human participants were reviewed and approved by Ethics Committee of Beijing HuiLongGuan Hospital. The patients/participants provided their written informed consent to participate in this study.

## Author Contributions

JXC and XJY designed the study, revised the manuscript, and wrote the final version. XJY and LG completed the design of the questionnaire. LBZ and JXC analyzed the data. SYZ, LGZ, and MQ have contributed to data collection. SJZ participated in the revising of the manuscript. JXC received funding support for the research. All authors contributed to the final draft of the manuscript.

## Funding

This work was supported by Capital Foundation of Medicine Research and Development (Grant Number: 2018-3-2132) and the Special Foundation of Beijing Municipal Science and Technology Commission (Grant Number: Z171100001017001).

## Conflict of Interest

The authors declare that the research was conducted in the absence of any commercial or financial relationships that could be construed as a potential conflict of interest.

## Publisher's Note

All claims expressed in this article are solely those of the authors and do not necessarily represent those of their affiliated organizations, or those of the publisher, the editors and the reviewers. Any product that may be evaluated in this article, or claim that may be made by its manufacturer, is not guaranteed or endorsed by the publisher.
