# Supplementary material for: The Professional Identity and Career Attitude of Chinese Medical Students During the COVID-19 Pandemic: A Cross-Sectional Survey in China
Source: Front Psychiatry. 2022 Feb 15;13:774467. doi: 10.3389/fpsyt.2022.774467 (PMC8886109; doi:10.3389/fpsyt.2022.774467)
Supplement: Supplementary file 1 [file Data_Sheet_1.docx]

Appendix A

Please answer to what extent you agree or disagree with the following statements. The response for each item is graded on a 5-point Likert scale: 1= Strongly Disagree 2= Somewhat Disagree 3=Neither Agree nor Disagree 4= Somewhat Agree 5= Strongly Agree

| 1 2 3 4 5 |
| --- |
| 1. I know the responsibility of a doctor. |
| 2. I am very proud of being a doctor. |
| 3. I am willing to engaged in the medical profession for my lifetime. |
| 4. I’d be happy to attend training and other activities which will help me become a doctor. |
| 5. I hope to achieve certain goals in my career as a doctor. |
| 6. I think being a doctor can realize the value of my life. |
